# A Robust Symbiotic Relationship Between the Ciliate *Paramecium multimicronucleatum* and the Bacterium *Ca*. Trichorickettsia Mobilis

**DOI:** 10.3389/fmicb.2020.603335

**Published:** 2020-11-24

**Authors:** Timofey Mironov, Elena Sabaneyeva

**Affiliations:** Department of Cytology and Histology, Biological Faculty, Saint-Petersburg State University, Saint-Petersburg, Russia

**Keywords:** symbiosis, ciliate, rickettsia, persistence, antibiotic, holobiont, cell wall deficient form, autophagy

## Abstract

Close reciprocal interactions in symbiotic systems have suggested the holobiont concept, in which the host and its microbiota are considered as a single entity. Ciliates are known for their ability to form symbiotic associations with prokaryotes. Relationships between the partners in such systems vary from mutualism to parasitism and differ significantly in their robustness. We assessed the viability of the ciliate *Paramecium multimicronucleatum* and its ability to maintain its intranuclear endosymbiont *Ca*. Trichorickettsia mobilis (Rickettsiaceae) after treatment with antibiotics characterized by different mode of action, such as ampicillin, streptomycin, chloramphenicol, tetracycline. The presence of endosymbionts in the host cell was determined by means of living cell observations made using differential interference contrast or fluorescence *in situ* hybridization with the species-specific oligonucleotide probe (FISH). Administration of antibiotics traditionally used in treatments of rickettsioses, tetracycline and chloramphenicol, depending on the concentration used and the ciliate strain treated, either caused death of both, infected and control cells, or did not affect the ability of the host to maintain the intranuclear endosymbiont. The surviving cells always manifested motile bacteria in the macronucleus. Streptomycin treatment never led to the loss of endosymbionts in any of the four infected strains, and nearly all ciliates remained viable. Ampicillin treatment never caused host cell death, but resulted in formation of filamentous and immobile oval bacterial forms. Under repeated ampicillin treatments, a part of endosymbionts was registered in the host cytoplasm, as evidenced both by FISH and transmission electron microscopy. Endosymbionts located in the host cytoplasm were enclosed in vacuoles, apparently, corresponding to autophagosomes. Nevertheless, the bacteria seemed to persist in this compartment and might cause relapse of the infection. Although the antibiotic sensitivity profile of Trichorickettsia seems to resemble that of other representatives of Rickettsiaceae, causative agents of severe diseases in humans, neither of the antibiotic treatments used in this study resulted in an aposymbiotic cell line, apparently, due to the protists’ sensitivity to tetracyclines, the drugs of preference in rickettsiosis treatment. The observed robustness of this symbiotic system makes it a good model for further elaboration of the holobiont concept.

## Introduction

Extensive studies of symbiotic systems occurring in nature and recognition of their importance in ecology and evolution has led to further elaboration of a holobiont concept first proposed by Margulis (1991). In the original meaning, a holobiont is a host with its inherited endosymbiont. According to the modern understanding, any organism together with its microbiota should be regarded as a single entity possessing a hologenome, the summarized genome of the host and its microbiota (Zilber-Rosenberg and Rosenberg, 2008; Bordenstein and Theis, 2015; Theis et al., 2016). The holobiont is prone to natural selection and can be considered as an evolutionary unit (Rosenberg and Zilber-Rosenberg, 2018). The holobiont concept, though eagerly accepted by many scholars (Bosch and Miller, 2016; O’Malley, 2017; Simon et al., 2019), has been criticized on the part of the adepts of the ecological approach to the studies of symbiotic systems (Moran and Sloan, 2015; Douglas and Werren, 2016; Foster et al., 2017) and hotly debated (Morris, 2018; Baedke et al., 2020). The main points of criticism concerned restrictions of the holobiont concept with regard to host fidelity and the way of symbiont transmission (Douglas and Werren, 2016). Indeed, it seems that the most important limitation of the holobiont concept is the stability of symbiotic relationship, since the system prone to easy changes of the partners could be hardly considered a single entity.

Symbiotic associations between ciliates and various pro- and eukaryotic microorganisms frequently occur in nature (Görtz and Fokin, 2009; Görtz, 2010; Fokin, 2012; Schweikert et al., 2013; Vannini et al., 2017; Castelli et al., 2019; Fokin et al., 2019; Flemming et al., 2020; Sonntag and Sommaruga, 2020). These associations demonstrate wide variability in partners’ fidelity and system stability, as well as both ways of the endosymbiont transmission, horizontal and vertical (Schweikert et al., 2013). Thus, symbiotic systems in ciliates represent a vast field for consideration of the holobiont concept (Serra et al., 2019). One of the best studied symbiotic systems involving ciliates, *Euplotes/Polynucleobacter necessarius*, for a long time has seemed a perfect model of a holobiont, demonstrating stability of the partnership, vertical transmission of the endosymbiont and apparent partner fidelity (Heckmann and Schmidt, 1987; Vannini et al., 2005, 2007). However, recent findings (Boscaro et al., 2018) have revealed replacement of the endosymbiotic bacteria in *Euplotes*, which calls into question regarding this system as a model holobiont subjected to evolutionary changes.

A peculiar flagellated endosymbiont *Ca*. Trichorickettsia mobilis (hereinafter referred to as *Ca*. T. mobilis) has been described in the macronucleus of the ciliate *Paramecium multimicronucleatum* (Vannini et al., 2014). This bacterium, so far, the only endosymbiont of ciliates characterized by incessant motility inside the host nucleus, belongs to Rickettsiaceae family, notorious mainly for some of its members—causative agents of serious human diseases, such as epidemic typhus (*Rickettsia prowazekii)* and Rocky Mountains spotted fever (*R. rickettsii*). Rickettsia are known to be resistant to many antibiotics (Rolain et al., 1998; Rolain, 2007; Walker, 2009; Fischer, 2018), moreover, *R. prowazekii* is known to persist in humans for a long time and cause sporadic relapse of the disease (Brill-Zinsser disease) in some patients many years after their recovery from epidemic typhus (Raoult and Roux, 1999; Weissmann, 2005; Sekeyová et al., 2019). Like all Rickettsiaceae, *Ca.* T. mobilis is characterized by an obligatory intracellular lifestyle. Our preliminary observations of *P. multimicronucleatum* strains bearing *Ca*. T. mobilis in their macronucleus—permanent 100% infection prevalence under different cultivation conditions including temperature shifts and starvation, which often cause loss of other endosymbionts in ciliates (Schweikert et al., 2013; Bella et al., 2016)—suggested the existence of a very stable relationship in *P. multimicronucleatum/Ca*. T. mobilis symbiotic system. So, although representatives of *Ca*. T. mobilis demonstrate intraspecies variability and the absence of the host and the compartment specificity (Sabaneyeva et al., 2018), *P. multimicronucleatum*/*Ca*. T. mobilis symbiotic system may represent a rare model of an ideal holobiont in the current sense of this term.

Our study pursued several objectives: to assess the stability of the symbiotic system *P*. *multimicronucleatum/Ca.* T. mobilis under treatments with antibiotics of different mechanism of action and the possibility of obtaining aposymbiotic cell lines, as well as to compare antibiotic susceptibility of intranuclear *Ca.* T. mobilis from the ciliates (Protista) to that of human pathogenic rickettsia. Therefore, among the antibiotics used were streptomycin, usually used for obtaining aposymbiotic cell lines in ciliates (Kusch et al., 2002; Dusi et al., 2014; Bella et al., 2016; Grosser et al., 2018) and ampicillin, applied in the former studies of motile intranuclear endosymbionts of *P. multimicronucleatum* (Vishnyakov and Rodionova, 1999), as well as chloramphenicol and tetracycline, which are the drugs of choice in treatments of rickettsioses in humans (Wisseman et al., 1974; Raoult, 1989; Rolain, 2007; Walker, 2009). The presence or absence of the endosymbiotic bacteria in the host cell after antibiotic administration was registered by means of living cell observations performed using differential interference contrast (DIC) and fluorescence *in situ* hybridization (FISH) with the species-specific oligonucleotide probe. The changes of the endosymbiont morphology and location upon ampicillin treatment were demonstrated with transmission electron microscopy (TEM). The last, but not the least, we proposed to answer the question whether association between these two microorganisms is robust enough to consider it a model holobiont.

## Materials and Methods

### Cell Cultures

*Paramecium multimicronucleatum* strains infected with the motile intranuclear endosymbiont *Ca*. Trichorickettsia mobilis used in the experiments are listed in Table 1.

**TABLE 1:** *Paramecium multimicronucleatum* strains infected with *Ca.* Trichorickettsia mobilis.

**Strain**	**Origin**	**Year of isolation**	**Collector**
LSA11-2	Lucca, Italy	2011	Sabaneyeva E. V.
Büsnau	Stuttgart, Germany	1988	Görtz H.-D.
ÀB9-4	Boston, United States	1994	Skoblo I. I.
Kr154-4	Krasnoyarsk, Russia	2012	Potekhin A. A.

All strains, except Büsnau strain, were maintained at RC CCM Culture Collection (Core Facility Center for Cultivation of Microorganisms) and were kindly provided by N. A. Lebedeva. Büsnau strain was a generous gift of Michael Schweikert (University of Stuttgart, Germany). The endosymbiont-free *P. multimicronucleatum* strains MSA and CyP5-3 from the same collection were used as controls to check the effect of the antibiotic on the host cells. The ciliates were maintained in tubes with the lettuce infusion inoculated with *Klebsiella aerogenes* at room temperature in the dark. Twice a week the cultures were fed by adding 1–2 mL of fresh bacterized medium. Prior to antibiotic administration the infection prevalence was assessed in all strains by means of fluorescence *in situ* hybridization (FISH) with the species-specific oligonucleotide probe.

### Antibiotic Treatment

Four antibiotics with different mode of action were used in the experiments: streptomycin (aminoglycoside class, inhibits bacterial protein synthesis) (Pagkalis et al., 2011); ampicillin (β-lactam class; inhibits bacterial cell wall synthesis) (Rafailidis et al., 2007); chloramphenicol (amphenicol class, inhibits bacterial protein synthesis) (Barnhill et al., 2012); tetracycline (tetracycline class, inhibits bacterial protein synthesis) (Chopra and Roberts, 2001). Stock solutions of streptomycin (Sigma-Aldrich, St. Louis, United States), ampicillin (Sigma-Aldrich, St. Louis, United States) and tetracycline (Sigma-Aldrich, St. Louis, United States) were prepared in distilled water, stock solutions of chloramphenicol (Sigma-Aldrich, St. Louis, United States)—in ethanol.

Fifty ciliates of each strain were placed in a microwell of a 24-microwell plate containing 0.5 mL of slightly bacterized lettuce infusion. In the first set of experiments, performed with all antibiotics, except tetracycline, an antibiotic was added to adjust the final concentration to 100, 250, 500, and 1,000 μg/mL. The microwell plates were kept in the dark at room temperature. The host cells were checked for viability and the presence of the endosymbionts on the 3rd, 10th, and 15th day after the treatment. After the first check 3–4 drops of bacterized culture medium were added twice a week to feed the ciliates. Since tetracycline proved to be extremely harmful for the host in the preliminary tests, tetracycline was administered in concentrations 10-fold lower than in the experiments with the other three antibiotics—10, 25, 50 and 100 μg/mL, and the results of this experiment were checked on the 3rd day. Besides that, we used an extra control endosymbiont free strain (CyP5-3) in the experiments with tetracycline. Each experiment (each concentration used with each strain) was repeated three times.

In another set of experiments, 1,000 μg/mL ampicillin was repeatedly (every 3rd day) administered to the microwell containing the same volume of the culture medium and the same number of Kr154-4 cells together with the bacterized culture medium. The ciliates were kept for 21 days. The cells were checked periodically for the host viability and the presence of endosymbionts. On the 5th (after the second ampicillin treatment) and on the 15th day of the experiment a part of the ciliates was fixed for TEM, and a part of the cells was fixed for FISH.

### Registration of Infection

The cell check for the infection was performed by living cell observations using Skovorodkin’s compression device (Skovorodkin, 1990) and a Leica 6000B microscope (Leica Microsystems, GmbH, Wetzlar, Germany) equipped with differential interference contrast (DIC), phase contrast and a digital camera DFC500. The culture was considered infected if five randomly chosen cells in a row manifested motile endosymbionts in their macronucleus. As it was impossible to obtain clear images of living bacteria due to their fast motility, for microphotography ciliates were fixed with 4% paraformaldehyde and observed with DIC. In some experiments, fluorescence *in situ* hybridization (FISH) using species-specific or group-specific probes was performed to ascertain the presence of various bacterial forms.

### Fluorescence *in situ* Hybridization (FISH)

The cells were fixed with cold 4% paraformaldehyde in 0.2M PBS in a microwell for 1 h and transferred to the Super Frost slides (Menzel-Gläser, Germany). The fixative was removed with a thin capillary and filter paper and 0.2M PBS was added to remove the remaining fixative solution. Then the buffer was removed and replaced with 70% methanol for 2–5 min. Changes of fixative and washes were controlled under a stereomicroscope. After a brief final wash in PBS, hybridization was carried out according to Manz and colleagues (Manz et al., 1992), at 30% formamide in the hybridization buffer at 46°C for 1.5 h. The species-specific probe RickFla_430 (5′-TCTTCCCTGCTAAAAGAACTTT-3; Vannini et al., 2014) labeled with Cy3 was used in combination with the almost universal bacterial probe Eub_338 (5′-GCTGCCTCCCGTAGGAGT-3′) labeled with FITC. Alternatively, in some cases the Alphaproteobacteria specific probe Alf_1b (5′-CGTTCGYTCTGAGCCAG-3′; Manz et al., 1992) labeled with Cy3 was used, which permitted to distinguish endocytobionts from the food bacteria *K. aerogenes* (Gammaproteobacteria). Hybridization was followed by two incubations in washing solution at 48°C, each for 30 min. The slides were mounted in Mowiol (Calbiochem, Germany) containing PPD and DAPI prepared according to manufacturer’s protocol. The slides were analyzed with a Leica TCS SPE Confocal Laser Scanning Microscope (CLSM). The images were processed with Fiji-win32 open access software (Schindelin et al., 2012).

### Transmission Electron Microscopy (TEM)

The cells were fixed in a mixture of 1.6% paraformaldehyde and 2.5% glutaraldehyde prepared with a phosphate buffer (0.1 M, pH 7.4) for 1.5 h at room temperature as described by Szokoli et al. (2016). Alternatively, 2.5% glutaraldehyde diluted with 0.1M cacodylate buffer (pH 7.4) was used as a fixative. The cells were washed in the same buffer containing 12.5% sucrose and postfixed in 1.6% OsO4 (1 h at 4°C). Then the cells were dehydrated in ethanol gradient followed by ethanol/acetone mixture (1:1), 100% acetone and embedded in Epoxy embedding medium (FlukaChemie AG, St. Gallen, Switzerland) according to the manufacturer’s protocol. The blocks were sectioned with a Leica EM UC6 Ultracut and ultrathin sections were stained with aqueous 1% uranyl acetate followed by 1% lead citrate. All samples were examined with a JEM-1400 electron microscope (JEOL Ltd., Tokyo, Japan) or JEM-2000 (JEOL Ltd., Tokyo, Japan) at 90 kV.

## Results

### Survival of Endosymbionts and Their Host Under Antibiotic Administration

**FIGURE 1:**
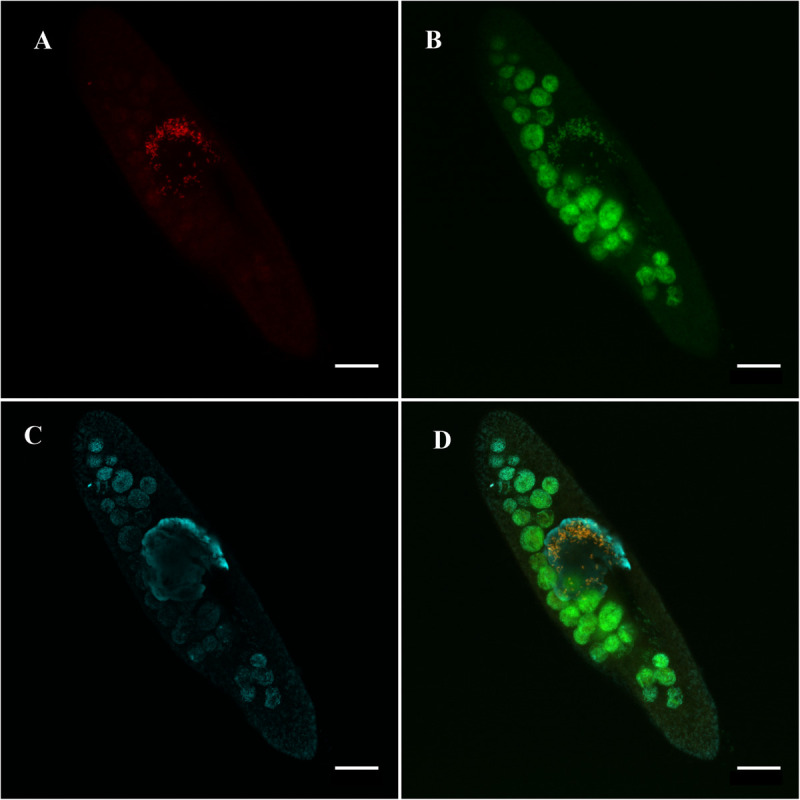
*Ca*. Trichorickettsia mobilis in the macronucleus of *Paramecium multimicronucleatum* (LSA11-2 strain) in the absence of antibiotics. Note exclusively intranuclear location of the endosymbiont. Fluorescence *in situ* hybridization, confocal laser scanning microscopy. **(A)** Species-specific probe RickFla_430 (Cy3, red signal). **(B)** Eubacterial probe Eub_338 (FITC, green signal). **(C)** Macronucleus is slightly counterstained with DAPI (cyan). **(D)** Merged image. Intranuclear bacteria revealed with both, the species-specific probe and the eubacterial probe are in yellow. Scale bar, 20 μm.

**FIGURE 2:**
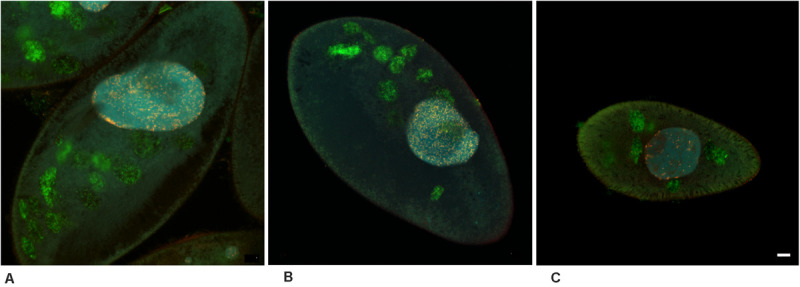
*Ca*. Trichorickettsia mobilis in the macronucleus of the three untreated *Paramecium multimicronucleatum* strains. Fluorescence *in situ* hybridization with the same probes, as in Figure 1. Confocal laser scanning microscopy. Endosymbionts demonstrate exclusively intranuclear location in all the three strains. **(A)** AB9-4. **(B)** Kr154-4. **(C)** Büsnau. Scale bar, 10 μm.

**FIGURE 3:**
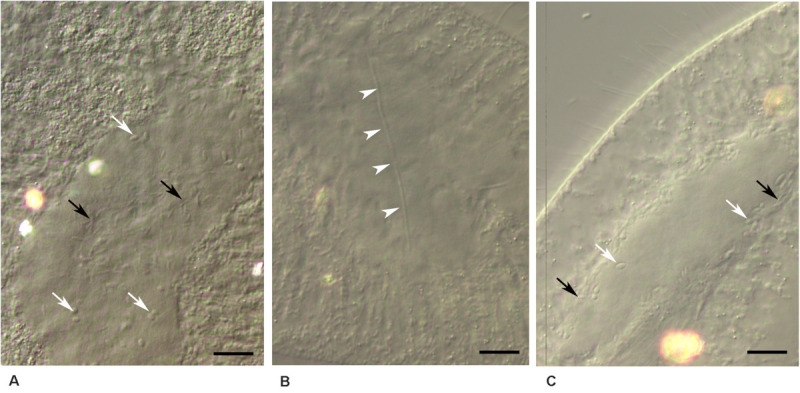
*Ca*. Trichorickettsia mobilis in the macronucleus of *Paramecium multimicronucleatum* after antibiotic administration. Unstained cells fixed with paraformaldehyde. DIC. **(A)** Short rod-like cells (black arrows), ovoid forms (white arrows); ampicillin, 1,000 μg/ml, 10th day. **(B)** Filamentous form is shown with the arrowheads; ampicillin, 500 μg/ml, 3rd day. **(C)** Ovoid forms (white arrows), short rod-like cells (black arrows); chloramphenicol, 500 μg/ml, 10th day. **(A,B)** Strain LSA11-2, **(C)** strain Kr154-4. Scale bar, 10 μm.

**FIGURE 4:**
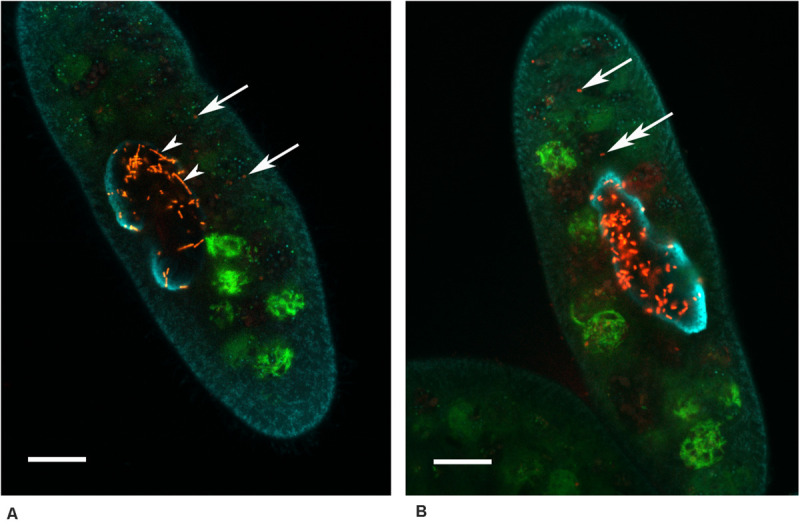
*Paramecium multimicronucleatum* (Kr154-4 strain) with the intranuclear *Ca*. Trichorickettsia mobilis on the 15th day of the repeated ampicillin treatment (1,000 μg/ml). Fluorescence *in situ* hybridization with the species-specific probe RickFla_430 (Cy3, red signal) and the universal eubacterial probe Eub_338 (Fluo, green signal), macronucleus slightly counterstained with DAPI (cyan) to see the nuclear contour. Confocal laser scanning microscopy. Some bacteria (arrow) can be seen outside the host macronucleus. **(A)** Filamentous forms (arrowheads) are seen in the macronucleus alongside with the short rod-shaped bacteria. **(B)** The bacterium marked with a two headed arrow resides in a vacuole devoid of food bacteria. Scale bar, 10 μm.

Prior to antibiotic treatments, the strains were checked for the presence of the endosymbionts using FISH experiments with the species-specific probe RickFla_430 (Vannini et al., 2014). All strains demonstrated 100% prevalence of infection, all endosymbiotic bacteria being located exclusively inside the macronucleus (Figures 1, 2). *In vivo* observations revealed high motility of the endosymbionts (not shown). Results of experimental treatments with streptomycin, ampicillin and chloramphenicol are summarized in Table 2. As seen from the table, streptomycin at all concentrations used affected neither viability of the host cells, nor motility or nuclear location of their endosymbionts. The only exception was the highest concentration (1,000 μg/mL) used with LSA11-2 and Büsnau strains, which resulted in the host cell death in both strains as early as by the 3rd day of the experiment. Administration of ampicillin induced transition of a subpopulation of bacteria from the typical motile short rod-shaped form to non-motile ovoid or filamentous forms in all tested strains, as shown with DIC (Figure 3) and FISH (Figure 4). Filamentous forms of bacteria could reach more than 50 μm long. In LSA11-2 and Büsnau strains, this effect was registered under all ampicillin concentrations including the lowest one (100 μg/mL), while in AB9-4 and Kr154-4 strains the filamentous forms were observed starting with 250 μg/mL concentration and ovoid forms were registered only under the highest concentration (1,000 μg/mL). Interestingly, treatment with ampicillin also caused the exit of the endosymbionts into the host cytoplasm, since in FISH experiments, besides the macronucleus, the positive, though faint signal of the species specific RickFla_430 probe was registered in the cytoplasm (Figure 4 and Supplementary Figure 1). Noteworthy, as a rule, the signal did not occur within the food vacuoles revealed with the nearly universal eubacterial probe Eub_338.

**TABLE 2:** Influence of streptomycin, ampicillin and chloramphenicol on *Paramecium multimicronucleatum/Ca*. Trichorickettsia mobilis symbiotic system.

**Drug**	**Concentration**	**100 μg/mL**			**250 μg/mL**			**500 μg/mL**			**1,000 μg/mL**		
	Strain/Day	3	10	15	3	10	15	3	10	15	3	10	15
Streptomycin	LSA11-2	+b	+b	+b	+b	+b	+b	+b	+b	+b	–	–	–
	Büsnau	+b	+b	+b	+b	+b	+b	+b	+b	+b	–	–	–
	Kr154-4	+b	+b	+b	+b	+b	+b	+b	+b	+b	+b	+b	+b
	AB9-4	+b	+b	+b	+b	+b	+b	+b	+b	+b	+b	+b	+b
	MSA (control)	+	+	+	+	+	+	+	+	+	+	+	+
Ampicillin	LSA11-2	+bf	+bf	+bf	+bf	+bfo	+bf	+bf	+bfo	+bf	+bfo	+bfo	+bfo
	Büsnau	+bf	+bfo	+bf	+bf	+bfo	+bf	+bf	+bfo	+bf	+bfo	+bfo	+bfo
	Kr154-4	+b	+b	+b	+bf	+bf	+bf	+bf	+bf	+bf	+bfo	+bfo	+bfo
	AB9-4	+b	+b	+b	+bf	+bf	+bf	+bf	+bf	+bf	+bfo	+bfo	+bfo
	MSA (control)	+	+	+	+	+	+	+	+	+	+	+	+
Chloramphenicol	LSA11-2	–	–	–	–	–	–	–	–	–	–	–	–
	Büsnau	–	–	–	–	–	–	–	–	–	–	–	–
	Kr154-4	+b	+b	+b	+b	+b	+b	+b	+bfo	+bfo	–	–	–
	AB9-4	+b	+b	+b	+b	+b	+b	+b	+bo	+b	–	–	–
	MSA (control)	+	+	+	+	+	+	+	+	+	–	–	–

**≪+≫, ciliates alive; ≪−≫, 100% ciliates dead; ≪b≫, motile bacteria observed in MA of ciliates; ≪o≫, ovoid forms of bacteria present; ≪f≫, filamentous forms present.**

Transition of the endosymbionts to ovoid and filamentous forms was also observed in Kr154-4 and AB9-4 strains treated with 500 μg/mL chloramphenicol (Table 2 and Figure 3C). However, the response of the strains to chloramphenicol differed significantly: this treatment caused host cell death in LSA11-2 and Büsnau strains under all concentrations used, whereas in Kr154-4 and AB9-4 strains the ciliates remained viable and maintained motile bacteria in the macronucleus under all concentrations, except for the highest one—1,000 μg/mL, which was lethal for the ciliates of non-infected MSA strain as well. This concentration caused the host cell death as early as in several hours after the start of the experiment.

Since Büsnau strain was lost in the course of time, the effect of tetracycline treatment was assessed using the three remaining strains—LSA11-2, Kr154-4, and AB9-4. All of these strains and the non-infected control strains MSA and CyP5-3 proved to be highly susceptible to tetracycline, which had to be used in the concentrations which were10-fold less, than those used with the three other antibiotics tested (Table 3). Only under 10 μg/mL tetracycline paramecia of all strains survived the treatment and maintained motile bacteria in the macronucleus. All higher concentrations (25, 50, and 100 μg/mL) were lethal to the host.

**TABLE 3:** Viability of *Paramecium multimicronucleatum* and intranuclear *Ca*. Trichorickettsia mobilis on the 3rd day after tetracycline administration.

**Strain/Concentration**	**10 μg/mL**	**25 μg/mL**	**50 μg/mL**	**100 μg/mL**
LSA11-2	+b	–	–	–
Kr154-4	+b	–	–	–
AB9-4	+b	–	–	–
MSA (control)	+	–	–	–
CYP5-3 (control)	+	–	–	–

**≪+≫, ciliates alive, ≪−≫, 100% ciliates dead, ≪b≫, motile bacteria observed in MA of ciliates.**

Noteworthy, in all our experimental treatments of all infected strains surviving hosts always manifested the presence of at least several motile bacteria in the macronucleus. On no occasion did we manage to obtain any aposymbiotic cell line.

### Fine Structure and Intracellular Location of Endosymbionts Under Ampicillin Treatment

**FIGURE 5:**
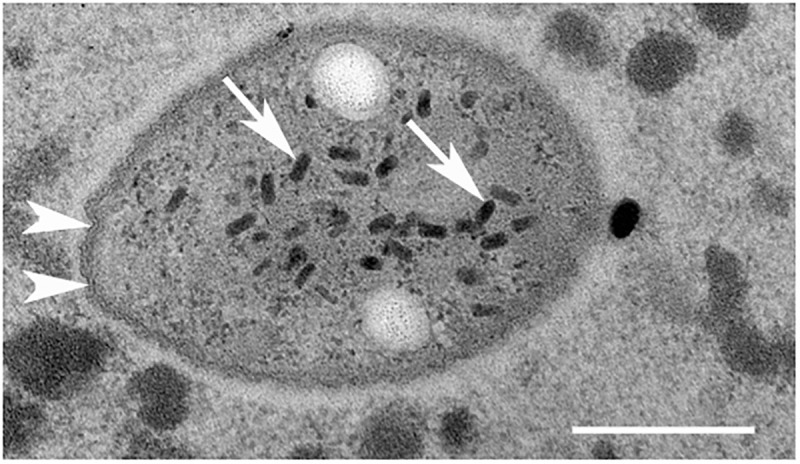
Transmission electron microscopy of *Ca*. Trichorickettsia mobilis in the macronucleus of *Paramecium multimicronucleatum* (strain Kr154-4) in the absence of antibiotic treatments. Note tightly adjacent membranes (white arrowheads) and virus-like particles (arrows) in the bacterial cells. Scale bar, 200 nm.

**FIGURE 6:**
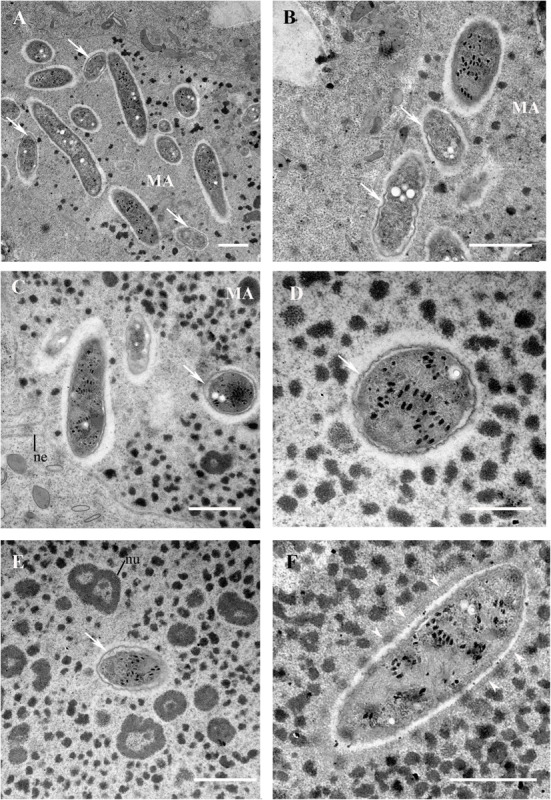
*Ca*. Trichorickettsia mobilis in the macronucleus of *Paramecium multimicronucleatum* (strain Kr154-4) after repeated ampicillin treatment. Transmission electron microscopy. **(A,B,F)** 5th day, **(C–E)** 15th day after the start of the experiment. **(A–E)** Bacterial cells with the detached outer membrane are marked with arrows. **(F)** A bacterium surrounded by a layer of fine fibrous material (arrowheads). MA, macronucleus; ne, nuclear envelope; nu, nucleoli. Scale bar in all plates, except **(D)** 1μm; in **(D)** 500 nm.

In the ciliates not exposed to any treatment, the outer and the plasma membranes of *Ca*. T. mobilis were usually rather closely apposed, and were often difficult to distinguish, regardless of the way of fixation (Figure 5). Neither were conspicuous delicate flagella, which are characteristic of this species and visualized best with the negative staining procedure (Vannini et al., 2014). At the same time, highly electron dense viral capsid-like particles were always visible. In the macronucleus of the ciliates treated with ampicillin, in the same cross-section, alongside with the bacteria possessing tightly adjacent outer and plasma membranes, some part of the endosymbionts demonstrated enlarged space between the two membranes, suggesting disorders in the bacterial cell wall (Figure 6A–E). The general appearance of the endosymbionts inside the host cell macronucleus did not change with time, bacterial cells with the detached outer membrane being registered on the 5th day after the start of the experiment, as well as on the 15th day. Some endosymbionts located in the macronucleus were surrounded by a thin layer of a fibrous material with electron density similar to that of the interchromatin compartment, but differing from the latter by regular arrangement (Figure 6F). Interestingly, in such bacterial cells the virus-like particles showed signs of disassembly, their outlines being less sharp, however, no perceptible detachment of the outer membrane of the bacterial cell was noted. In the TEM images, we did not come across longitudinal sections of *Ca*. T. mobilis (Figure 6), thus, it was not possible to distinguish filamentous forms from the rod-shaped forms with certainty.

**FIGURE 7:**
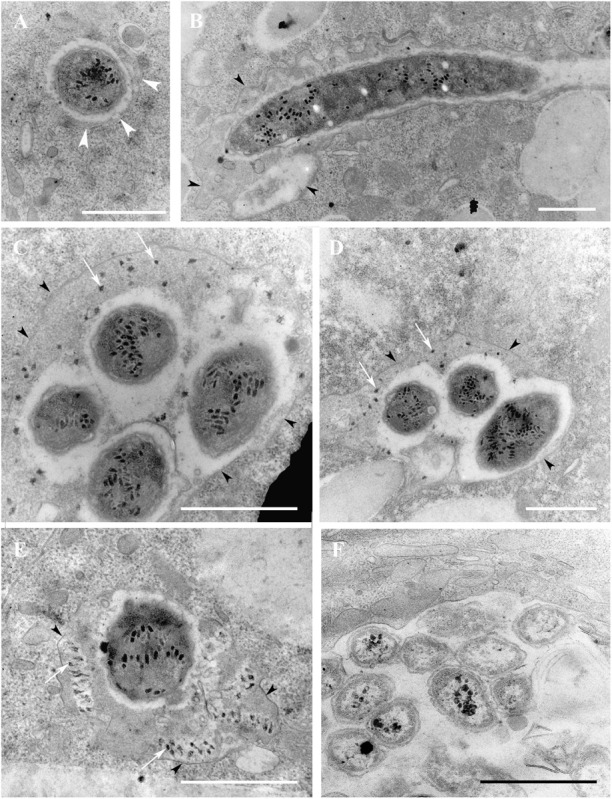
*Paramecium multimicronucleatum* (strain Kr154-4) on the 5th day after repeated ampicillin treatment. Transmission electron microscopy. **(A)** *Ca*. Trichorickettsia mobilis lying free in the host cytoplasm. Note a phagophore-like cisterna (shown with the white arrowheads) close to the endosymbiont. **(B)** Oblique section. Part of the filamentous form is seen enclosed in a vacuole (black arrowheads). **(C–E)** Cross section. *Ca*. Trichorickettsia mobilis inside the vacuole in the host cytoplasm. The membrane of the vacuole is indicated with black arrowheads. White arrows point to the virus-like particles outside the bacteria. **(F)** Food bacteria *Klebsiella aerogenes* in the food vacuole. Scale bar, 1 μm.

The TEM study confirmed our observations of bacterial egress from the host macronucleus upon ampicillin treatment made with FISH. The bacterial forms released from the host macronucleus only occasionally lay “naked” in the host cytoplasm (Figure 7A), sometimes, with a fine horseshoe-like cistern resembling a phagophore found in their vicinity. However, most of the endosymbiotic bacteria residing in the cytoplasm of ampicillin treated ciliates were located inside the vacuoles bounded by a single membrane (B–E). In some cases, lysosomes were found in the close proximity to the endosymbiont containing vacuole and seemed to fuse with it (Figure 7B). “Naked” *Ca*. T. mobilis and most of the endosymbionts enclosed in the cytoplasmic vacuoles looked very much the same as those located inside the macronucleus, however, the virus-like particles of somewhat lower electron density were observed outside the bacterial cells inside the host vacuoles (Figures 7C,D). The contents of these vacuoles were of medium electron density, comparable with the contents of lysosomes. Noteworthy, the endosymbionts inside these vacuoles were always surrounded by an electron lucid area, presumably resulting from the numerous poorly preserved bacterial flagella. In rare cases the virus-like particles underwent disassembly, while the endosymbiont manifested blebbing of its surface (Figure 7E). Thus, the general appearance of the endosymbiont containing vacuoles (Figures 7A–E) differed significantly from the newly formed phagosomes with the food bacteria (Figure 6F). The former contained exclusively *Ca*. T. mobilis, which was easily recognizable by the presence of the virus-like particles (Figures 7A–E), and never included any food bacteria. On the contrary, the phagosomes were characterized by electron lucid contents, and they enclosed only the food bacteria (Figure 7F).

## Discussion

### Stability of the Symbiotic System *P*. *multimicronucleatum/Ca.* T. Mobilis After Antibiotic Administration

Symbiotic system *Paramecium multimicronucleatum*/*Ca*. Trichorickettsia mobilis proved to be extremely stable under antibiotic treatments. Streptomycin, the antibiotic of the aminoglycoside group with a wide range of action, which is often used for obtaining aposymbiotic cell lines in ciliates (Grosser et al., 2018), was absolutely ineffective. This observation is in good agreement with the data on resistance of pathogenic rickettsia to aminoglycosides (Rolain and Raoult, 2005). Also, *Ca*. T. mobilis demonstrated insusceptibility to ampicillin, one of the penicillin group of antibiotics. This is not very surprising taking into account resistance of pathogenic rickettsia to the beta-lactam antibiotics (Rolain et al., 1998). Contrary to our expectations, we did not manage to obtain aposymbiotic cells with antibiotics used in clinical practice for the treatment of rickettsioses, either. Exposure to high concentrations of chloramphenicol turned out to be harmful for ciliates, which is in accordance with the general notion of the high toxicity of this drug (Shukla et al., 2011), presumably, affecting protein synthesis in the host mitochondria (Barnhill et al., 2012). Our failure to obtain endosymbiont-free cells after tetracycline administration is apparently due to high susceptibility of the host cells to tetracycline. The spectrum of activity of this group of antibiotics is known to encompass some parasitic protists, such as *Plasmodium falciparum*, *Giardia lamblia*, *Trichomonas vaginalis*, and some others (Chopra and Roberts, 2001). Although molecular mechanism of the antiparasitic effect of tetracyclines in protists remains unknown, the effect in some of them, e. g., *P. falciparum*, has been proposed to be caused by the inhibition of the protein synthesis in their mitochondria (Chopra and Roberts, 2001). Our data suggest that *P. multimicronucleatum* falls in the list of eukaryotes susceptible to tetracycline treatment as well. Possibly, tetracycline affects protein synthesis in the mitochondria of this species. Surprisingly, representatives of the same genus, *P. primaurelia* and *P. pentaurelia*, seem not to be susceptible to tetracycline, as aposymbiotic cell lines have been obtained with higher concentrations of this antibiotic while clearing the cells from *Ca*. Megaira polyxenophila (Pasqualetti et al., 2020).

In general, antimicrobial susceptibility testing in obligate intracellular bacteria is a challenging task, since it is limited by a range of the applicable tests (Vanrompay et al., 2017). It is only determining the minimal inhibitory concentration (MIC value) that is possible for uncultivable bacterial strains. Moreover, estimation of the final effective antibiotic concentration still remains elusive, as the concentration inside the host can differ from that in the culture medium (Wisseman et al., 1982). Besides that, the half-life of the particular antibiotic should differ depending on peculiarities of the host cell metabolism. Therefore, for the same intracellular bacterium, e.g., *Chlamydia*, MICs may differ in different cell lines (Suchland et al., 2003). In our study, we observed the differences of the ciliate strains (LSA11-2 and Büsnau *versus* Kr154-4 and AB9-4) in their response to administration of chloramphenicol and the highest concentration of streptomycin. The reason for higher vulnerability of the two strains (LSA11-2 and Büsnau) remains unclear. It might be connected either with the strain differences at the level of gene transcription and protein modifications, or, alternatively, it could be another example of intraspecies variability at the genome level, like the one we observed in *Paramecium calkinsi* infected with cytoplasmic *Ca*. T. mobilis (Sabaneyeva et al., 2018).

Overall, the antibiotic resistance pattern of *Ca*. T. mobilis seems to be very similar to that of pathogenic rickettsia, however, we did not manage to expel the endosymbionts by administering antibiotics used as a first-line treatment of rickettsioses. All our experiments led to two possible outcomes: either the host survived together with its endosymbiont, or the host died. Apparently, in the latter case the death of the host was accompanied by the death of the endosymbiotic bacteria. There could be two possible explanations of these data. On the one hand, it is tempting to suggest that the death of the ciliate could be caused by the death of its endosymbionts upon administration of tetracycline or a high dosage of chloramphenicol. This could mean that the ciliate might be dependent on viability and persistence of *Ca*. T. mobilis in its macronucleus. On the other hand, we cannot exclude another option—that tetracycline and high concentration of chloramphenicol may be harmful for the host itself, possibly, by affecting its mitochondria. Be that as it may, so far, it seems impossible to separate the partners of the symbiotic system *P. multimicronucleatum/Ca*. Trichorickettsia mobilis, which can be regarded as an ideal model holobiont in both, original and modern, senses of this term, as it not only represents a host with an inherited endosymbiont, but appears as a real single entity demonstrating significant stability under antibiotic pressure.

### Release of the Intranuclear Bacteria Into the Host Cytoplasm

A thin layer of a fibrous material surrounding some of the bacterial cells in the host nucleus looks very much like the one seen around *Holospora obtusa* in the macronucleus of the non-specific host *P. multimicronucleatum* under experimental infection under nocodazole treatment before the bacteria are expelled from the macronucleus (Fokin et al., 2005). This layer has been proposed to play a role in the extrusion of bacteria from the nucleus, however, it is not clear whether this is a feature typical only for *P. multimicronucleatum*, or it is a more general cellular mechanism for extrusion of some material from the nucleus. Interestingly, a similar layer was registered around the infectious forms of two other endonucleobionts residing in the macronucleus of two other species of paramecia—*Holospora curvata* from *P. calkinsi* and *Holospora bacillata* from *P. woodruffi* (Fokin and Sabaneyeva, 1997). In this case, the fibrillar layer has been also believed to be connected with the release of the endosymbionts from the host macronucleus. Thus, formation of such layer seems to be typical at least for *Paramecium* species. The composition of this layer remains enigmatic.

### Formation of Persister Cells Upon Treatment With Ampicillin and Chloramphenicol

Transition of motile intranuclear short rod-shaped bacteria to filamentous and ovoid forms, which occurred after ampicillin treatment in all tested strains, confirms the data of Vishnyakov and Rodionova (1999), who had reported on similar transitions in a motile intranuclear endosymbiont inhabiting the macronucleus of several strains of *P. multimicronucleatum.* Although the description of this endosymbiont lacked molecular characterization, on the basis of similar morphology and peculiar behavior, it seemed most likely, that the endosymbiont described in this study was no other than *Ca*. T. mobilis. Importantly, the strain AB9-4, which we used in our experiments and proved to harbor *Ca*. T. mobilis by FISH with the species-specific probe RickFla_430, was among the strains described by Vishnyakov and Rodionova. It is remarkable, that this strain has been maintained in the culture collection RC CCM (Core Facility Centre for Cultivation of Microorganisms, Saint-Petersburg State University) for more than 25 years. The strain has not lost its endosymbionts throughout a quarter of a century, which is another argument for the robustness of symbiotic relationship in *Ca*. T. mobils/*P. multimicronucleatum* system.

Since beta-lactam antibiotics inhibit the cell wall synthesis, formation of filamentous (or septate, as they were called by Vishnyakov and Rodionova) and ovoid bacterial cells must have been caused by disorders in the bacterial cell wall. Extension of the periplasmic space seen in some bacteria with TEM supports this idea, suggesting that ampicillin treatment induces formation of the cell wall deficient forms in *Ca*. T. mobilis. Noteworthy, formation of spheroplasts upon G-penicillin treatment has been reported in *R. prowazekii* (Wisseman et al., 1982), which can be sustained in the organism for a long time and can cause relapse of the infection (Raoult and Roux, 1999; Weissmann, 2005; Sekeyová et al., 2019). The recurrent diseases are generally believed to be caused not by reinfection, but by populations of persister cells, i.e., quiescent forms of bacteria (Fauvart et al., 2011; Brauner et al., 2016; Van den Bergh et al., 2016; Levin-Reisman et al., 2017; Trastoy et al., 2018; Mickiewicz et al., 2019). Most often persisters are represented by cell wall deficient forms (sometimes referred to as L-forms), which are believed to cause relapse of the disease long time after the patients’ recovery due to antibiotic administration (Onwuamaegbu et al., 2005; Mickiewicz et al., 2019). Transition to L-form state can be induced by treatments of cells with antibiotics affecting cell wall synthesis (Mercier et al., 2014; Errington et al., 2016). Cell wall deficient forms have been reported periodically in a wide range of bacteria, from *Mycoplasma tuberculosis* (Slavchev et al., 2016) to uropathogenic *Escherichia coli* (Mickiewicz et al., 2019).

Although the mechanism of *R. prowazekii* latency has not been definitely established (Sekeyová et al., 2019), possibly, it is persisting spheroplasts that serve as a source of recrudescence. Interestingly, recurrence of other rickettsia-borne diseases, such as Mediterranean spotted fever and murine typhus, has been registered even after treatment with chloramphenicol (Shaked et al., 1989). Disease relapses and persistence of the closely related to rickettsia species *Orientia tsutsugamushi* (scrub typhus rickettsiae) in tissues of recovered patients have been reported after both, chloramphenicol and doxycycline therapy (Kelly et al., 2017). Our data on appearance of ovoid forms upon exposure to sublethal concentrations of chloramphenicol in the strains Kr154-4 and AB9-4 seem to be in line with these observations. It is very likely, that the ovoid forms of *Ca*. T. mobilis correspond to spheroplasts of *R. prowazekii* and should be considered as persisters, which we believe to be responsible for stability of the symbiotic system *P. mutimicronucleatum*/*Ca*. T. mobilis. The faint signal produced in FISH experiments by the endosymbionts located in the host cytoplasm (Figure 4) may be caused by a low level of ribosome synthesis in the dormant persister cells, induced by antibiotic treatment.

Interestingly, *Ca*. T. mobilis seems to combine features characteristic of two groups of *Rickettsia*: intranuclear location, which has been registered for spotted fever group (SFG) rickettsia, and spheroplast transition, so far proposed only for *Rickettsia prowazekii*, belonging to typhus group (TG).

### The Nature of the Endosymbiont-Containing Vacuoles in Ampicillin Treated Cells

Exposure of the symbiotic system to ampicillin leads to the release of some endosymbionts into the host cytoplasm. Our detection of a phagophore-like structure closing on a “naked” endosymbiont in the host (Figure 7A) and of lysosomes fusing with the membrane of the endosymbiont containing vacuole (Figure 7B) suggests an autophagy-related process. The vacuoles in the host cytoplasm containing endosymbionts might be autophagolysosomes, since they possess one membrane and their contents seem to be similar to that of the lysosomes. It is not clear whether the released endosymbionts survive. The presence of virus-like particles outside the bacterial cells may be considered as signs of bacterial degradation, however, only in rare cases did we observe blebbing of the bacterial membrane (Figure 7E), reminding of the images interpreted as L-form proliferation (Errington, 2013). However, most of the bacteria seemed to remain intact, which is in good agreement with the recent finding that *Ca*. T. mobilis can survive in the planarian enterocytes for 7 days (Modeo et al., 2020). We suppose that egress of *Ca*. T. mobilis from the macronucleus into the host cytoplasm could trigger autophagy, a mechanism used to clear cells from alien microorganisms. Although in general autophagy is an antipathogenic process, many intracellular pathogens manage to develop means of escape from the host autophagosome or to hijack this dangerous compartment for their survival and propagation (Siqueira et al., 2018; Khandia et al., 2019). Some of them, e.g., *Shigella flexneri* or *Salmonella typhimurium*, inhibit the host autophagy machinery to prevent xenophagy, while others, like *Ehrlichia chaffeensis*, induce autophagy to obtain host cytosolic nutrients without running the risk of autophagic clearance (Jiao and Sun, 2019). Thus, a representative of the sister to Rickettsiaceae family Anaplasmataceae, *Anaplasma phagocytophilum*, subverts early autophagosomes of the host cell to facilitate bacterial proliferation (Niu et al., 2008). Some of the pathogens, such as *Porphyromonas gingivalis* or *Brucella* adapt to survival in the autophagosome by preventing its fusion with the host lysosomes (Khandia et al., 2019), whereas *Coxiella brunetti* is known to recruit autophagy related proteins to its replicative vacuole, and inhibition of autophagy leads to impairment of replication in *Coxiella* (Pareja et al., 2017). Keeping this in mind, we cannot exclude the possibility that *Ca*. T. mobilis, likewise, might be able to manipulate the host cell autophagy to ensure its survival. Another option to be considered is that *Ca*. T. mobilis might use autophagy as a host sparing way for egress from the host cell, like *Brucella*, *Mycobacterium marinum* and, possibly, *Francisella* (Friedrich et al., 2012).

To summarize, we showed that *Paramecium multimicronuclea -tum*/*Ca*. Trichorickettsia mobilis symbiotic system is very stable and its partners are inseparable under conventional antibiotic treatments. This peculiarity makes the system a good candidate for a model holobiont, which can be used in further elaboration of the holobiont concept. Stability of the system is most probably ensured by the ability of the endosymbiont to produce dormant persisters in the presence of ampicillin and chloramphenicol, a feature uniting them with pathogenic *R*. *prowazekii.* Ovoid and filamentous forms of *Ca*. T. mobilis might be subsequently investigated to elucidate the issue of bacterial persistence. Moreover, we propose that autophagy might be involved in the survival of *Ca*. T. mobilis released from the host macronucleus into the cytoplasm, and analyzing molecular mechanisms affected by the endosymbiont under administration of certain antibiotics might enrich our knowledge of bacterial-host crosstalk in the symbiotic system under destabilizing conditions.

## Data Availability Statement

The original contributions presented in the study are included in the article/Supplementary Material, further inquiries can be directed to the corresponding author.

## Author Contributions

ES designed the research. TM performed all the experiments. ES and TM interpreted the results. ES wrote the manuscript with contributions by TM. ES financed the research. Both authors critically read and approved the manuscript.

## Conflict of Interest

The authors declare that the research was conducted in the absence of any commercial or financial relationships that could be construed as a potential conflict of interest.

## Supplementary Material

Supplementary Figure 1 *Paramecium multimicronucleatum* (strain LSA11-2) on the 3rd day after ampicillin treatment. FISH with Alphaproteobacteria specific probe Alf_1b (red signal). Arrows point to *Ca*. Trichorickettsia mobilis located in the macronucleus (counterstained with DAPI, shown in white) and in the cytoplasm. Scale bar, 20 μm.

## References

[B1] Baedke J. Fábregas-Tejeda A. Delgado A. N. (2020). The holobiont concept before Margulis. *J. Exp. Zool.* 334 149–155. 10.1002/jez.b.22931 32039567

[B2] Barnhill A. E. Brewer M. T. Carlson S. A. (2012). Adverse effects of antimicrobials via predictable or idiosyncratic inhibition of host mitochondrial components. *Antimicrob. Agents Chemother.* 56 4046–4051. 10.1128/aac.00678-12 22615289 PMC3421593

[B3] Bella C. Koehler L. Grosser K. Berendonk T. U. Petroni G. Schrallhammer M. (2016). Fitness impact of obligate intranuclear bacterial symbionts depends on host growth phase. *Front. Microbiol.* 7:2084. 10.3389/fmicb.2016.02084 28066397 PMC5177645

[B4] Bordenstein S. R. Theis K. R. (2015). Host biology in light of the microbiome: ten principles of holobionts and hologenomes. *PLoS Biol.* 13:e1002226. 10.1371/journal.pbio.1002226 26284777 PMC4540581

[B5] Boscaro V. Fokin S. Petroni G. Verni F. Keeling P. Vannini C. (2018). Symbiont replacement between bacteria of different classes reveals additional layers of complexity in the evolution of symbiosis in the ciliate *Euplotes*. *Protist* 169 43–52. 10.1016/j.protis.2017.12.003 29414319

[B6] Bosch T. Miller D. (2016). *The Holobiont Imperative.* Vienna: Springer-Verlag 10.1007/978-3-7091-1896-2

[B7] Brauner A. Fridman O. Gefen O. Balaban N. Q. (2016). Distinguishing between resistance, tolerance and persistence to antibiotic treatment. *Nat. Rev. Microbiol.* 14 320–330. 10.1038/nrmicro.2016.34 27080241

[B8] Castelli M. Serra V. Senra M. V. X. Basuri C. K. Soares C. A. G. Fokin S. I. (2019). The hidden world of *Rickettsiales* symbionts: “*Candidatus* Spectririckettsia obscura”, a novel bacterium found in Brazilian and Indian *Paramecium caudatum*. *Microb. Ecol.* 77 748–758. 10.1007/s00248-018-1243-8 30105505

[B9] Chopra I. Roberts M. (2001). Tetracycline antibiotics: mode of action, applications, molecular biology, and epidemiology of bacterial resistance. *Microbiol. Mol. Biol. Rev.* 65 232–260. 10.1128/mmbr.65.2.232-260.2001 11381101 PMC99026

[B10] Douglas A. E. Werren J. H. (2016). Holes in the hologenome: why host-microbe symbioses are not holobionts. *mBio* 7:e02099-15. 10.1128/mBio.02099-15 PMC4817262 27034285

[B11] Dusi E. Krenek S. Schrallhammer M. Sachse R. Rauch G. Kaltz O. (2014). Vertically transmitted symbiont reduces host fitness along temperature gradient. *J. Evol. Biol.* 27 796–800. 10.1111/jeb.12336 24779056

[B12] Errington J. (2013). L-form bacteria, cell walls and origins of life. *Open Biol.* 3:120143. 10.1098/rsob.120143 23303308 PMC3603455

[B13] Errington J. Mickiewicz K. Kawai Y. Wu L. J. (2016). L-form bacteria, chronic diseases and the origins of life. *Philos. Trans. R. Soc. Lond. B Biol. Sci.* 371:20150494. 10.1098/rstb.2015.0494 27672147 PMC5052740

[B14] Fauvart M. De Groote V. N. Michiels J. (2011). Role of persister cells in chronic infections: clinical relevance and perspectives on anti-persister therapies. *J. Med. Microbiol.* 60 699–709. 10.1099/jmm.0.030932-0 21459912

[B15] Fischer M. (2018). Rickettsioses: cutaneous findings frequently lead to diagnosis – a review. *J. Dtsch. Dermatol. Ges.* 16 1459–1476. 10.1111/ddg.13712 30537329

[B16] Flemming F. E. Potekhin A. Pröschold T. (2020). Algal diversity in *Paramecium bursaria*: species identification, detection of *Choricystis parasitica*, and assessment of the interaction specificity. *Diversity* 12:287 10.3390/d12080287

[B17] Fokin S. I. (2012). Frequency and biodiversity of symbionts in representatives of the main classes of Ciliophora. *Eur. J. Protistol.* 48 138–148. 10.1016/j.ejop.2011.12.001 22425549

[B18] Fokin S. I. Sabaneyeva E. V. (1997). Release of endonucleobiotic bacteria *Holospora bacillata* and *Holospora curvata* from the macronucleus of their host cells *Paramecium woodruffi* and *Paramecium calkinsi*. *Endocytob. Cell Res.* 12 49–55.

[B19] Fokin S. I. Schweikert M. Fujishima M. (2005). Recovery of the ciliate *Paramecium multimicronucleatum* following bacterial infection with *Holospora obtusa*. *Eur. J. Protistol.* 41 129–138. 10.1016/j.ejop.2004.11.007

[B20] Fokin S. I. Serra V. Ferrantini F. Modeo L. Petroni G. (2019). “*Candidatus* Hafkinia simulans” gen. nov., sp. nov., a novel *Holospora*-like bacterium from the macronucleus of the rare brackish water ciliate *Frontonia salmastra* (Oligohymenophorea, Ciliophora): multidisciplinary characterization of the new endosymbiont and its host. *Microb. Ecol.* 77 1092–1106. 10.1007/s00248-018-1311-0 30627761

[B21] Foster K. Schluter J. Coyte K. Rakoff-Nahoum S. (2017). The evolution of the host microbiome as an ecosystem on a leash. *Nature* 548 43–51. 10.1038/nature23292 28770836 PMC5749636

[B22] Friedrich N. Hagedorn M. Soldati-Favre D. Soldati T. (2012). Prison break: pathogens’ strategies to egress from host cells. *Microbiol. Mol. Biol. Rev.* 76 707–720. 10.1128/mmbr.00024-12 23204363 PMC3510522

[B23] Görtz H.-D. (2010). Microbial infections in free-living protozoa. *Crit. Rev. Immunol.* 30 95–106. 10.1615/critrevimmunol.v30.i1.70 20370623

[B24] Görtz H.-D. Fokin S. I. (2009). “Diversity of endosymbiotic bacteria in paramecium,” in *Endosymbionts in Paramecium*, ed. Fujishima M. (Berlin: Springer), 131–160. 10.1007/978-3-540-92677-1_6

[B25] Grosser K. Ramasamy P. Amirabad A. D. Schulz M. H. Gasparoni G. Simon M. (2018). More than the “killer trait”: infection with the bacterial endosymbiont *Caedibacter taeniospiralis* causes transcriptomic modulation in *Paramecium* host genome. *Biol. Evol.* 10 646–656. 10.1093/gbe/evy024 29390087 PMC5814942

[B26] Heckmann K. Schmidt H. J. (1987). *Polynucleobacter necessarius* gen. nov., sp. nov., an obligately endosymbiotic bacterium living in the cytoplasm of *Euplotes aediculatus*. *Int. J. Syst. Bacteriol.* 37 456–457. 10.1099/00207713-37-4-456

[B27] Jiao Y. Sun J. (2019). Bacterial manipulation of autophagic responses in infection and inflammation. *Front. Immunol.* 10:2821. 10.3389/fimmu.2019.02821 31849988 PMC6901625

[B28] Kelly D. J. I. Fuerst P. A. Richards A. L. (2017). The historical case for and the future study of antibiotic-resistant scrub typhus. *Trop. Med. Infect. Dis.* 2:E63. 10.3390/tropicalmed2040063 30270920 PMC6082054

[B29] Khandia R. Dadar M. Munjal A. Dhama K. Karthik K. Tiwari R. (2019). A comprehensive review of autophagy and its various roles in infectious, non-infectious, and lifestyle diseases: current knowledge and prospects for disease prevention, novel drug design, and therapy. *Cells* 8:674. 10.3390/cells8070674 31277291 PMC6678135

[B30] Kusch J. Czubatinski L. Wegmann S. Hübner M. Alter M. Albrecht P. (2002). Competitive advantages of *Caedibacter*-infected paramecia. *Protist* 153 47–58. 10.1078/1434-4610-00082 12022275

[B31] Levin-Reisman I. Ronin I. Gefen O. Braniss I. Shoresh N. Balaban N. (2017). Antibiotic tolerance facilitates the evolution of resistance. *Science* 355 826–830. 10.1126/science.aaj2191 28183996

[B32] Manz W. Amann R. Ludwig W. Wagner M. Schleifer K.-H. (1992). Phylogenetic oligodeoxynucleotide probes for the major subclasses of *Proteobacteria*: problems and solutions. *Syst. Appl. Microbiol.* 15 593–600. 10.1016/s0723-2020(11)80121-9

[B33] Margulis L. (1991). “Symbiogenesis and symbionticism,” in *Symbiosis as a Source of Evolutionary Innovation: Speciation and Morphogenesis*, eds Margulis L. Fester R. (Cambridge, MA: MIT Press), 1–14. 11538111

[B34] Mercier R. Kawai Y. Errington J. (2014). General principles for the formation and proliferation of a wall-free (L-form) state in bacteria. *eLife* 3:e04629. 10.7554/eLife.04629 PMC4244569 25358088

[B35] Mickiewicz K. Kawai Y. Drage L. Gomes M. Davison F. Pickard R. (2019). Possible role of L-form switching in recurrent urinary tract infection. *Nat. Commun.* 10:4379. 10.1038/s41467-019-12359-3 31558767 PMC6763468

[B36] Modeo L. Salvetti A. Rossi L. Castelli M. Szokoli F. Krenek S. (2020). “*Candidatus* Trichorickettsia mobilis”, a *Rickettsiales* bacterium, can be transiently transferred from the unicellular eukaryote *Paramecium* to the planarian *Dugesia japonica*. *PeerJ* 8:e8977. 10.7717/peerj.8977 32351785 PMC7183750

[B37] Moran N. A. Sloan D. B. (2015). The hologenome concept: helpful or hollow? *PLoS Biol*. 13:e1002311. 10.1371/journal.pbio.1002311 26636661 PMC4670207

[B38] Morris J. J. (2018). What is the hologenome concept of evolution? *F1000Res.* 7:F1000 Faculty Rev-1664. 10.12688/f1000research.14385.1 30410727 PMC6198262

[B39] Niu H. Yamaguchi M. Rikihisa Y. (2008). Subversion of cellular autophagy by *Anaplasma phagocytophilum*. *Cell. Microbiol.* 10 593–605. 10.1111/j.1462-5822.2007.01068.x 17979984

[B40] O’Malley M. A. (2017). From endosymbiosis to holobionts: evaluating a conceptual legacy. *J. Theor. Biol.* 434 34–41. 10.1016/j.jtbi.2017.03.008 28302492

[B41] Onwuamaegbu M. E. Belcher R. A. Soare C. (2005). Cell wall-deficient bacteria as a cause of infections: a review of the clinical significance. *J. Int. Med. Res.* 33 1–20. 10.1177/147323000503300101 15651712

[B42] Pagkalis S. Mantadakis E. Mavros M. N. Ammari C. Falagas M. E. (2011). Pharmacological considerations for the proper clinical use of aminoglycosides. *Drugs* 71 2277–2294. 10.2165/11597020-000000000-00000 22085385

[B43] Pareja M. E. M. Bongiovanni A. Lafont F. Colombo M. I. (2017). Alterations of the *Coxiella burnetii* replicative vacuole membrane integrity and interplay with the autophagy pathway. *Front. Cell. Infect. Microbiol.* 7:112. 10.3389/fcimb.2017.00112 28484683 PMC5401879

[B44] Pasqualetti C. Szokoli F. Rindi L. Petroni G. Schrallhammer M. (2020). The obligate symbiont “*Candidatus* Megaira polyxenophila” has variable effects on the growth of different host species. *Front. Microbiol.* 11:1425 10.3389/fmicb.2020.01425 PMC7360802 32733401

[B45] Rafailidis P. I. Ioannidou E. N. Falagas M. E. (2007). Ampicillin/sulbactam current status in severe bacterial infections. *Drugs* 67 1829–1849. 10.2165/00003495-200767130-00003 17722953

[B46] Raoult D. (1989). Antibiotic susceptibility of *Rickettsia* and treatment of rickettsioses. *Eur. J. Epidemiol.* 5 432–435. 10.1007/bf00140135 2606171

[B47] Raoult D. Roux V. (1999). The body louse as a vector of reemerging human diseases. *Clin. Infect. Dis.* 29 888–911. 10.1086/520454 10589908

[B48] Rolain J. M. (2007). “Antimicrobial susceptibility of rickettsial agents,” in *Rickettsial Diseases*, eds Raoult D. Parola P. (New York, NY: Informa Healthcare), 361–369. 10.3109/9781420019971.026

[B49] Rolain J. M. Maurin M. Vestris G. Raoult D. (1998). In vitro susceptibilities of 27 rickettsiae to 13 antimicrobials. *Antimicrob. Agents Chemother*. 42 1537–1541. 10.1128/aac.42.7.1537 9660979 PMC105641

[B50] Rolain J. M. Raoult D. (2005). Prediction of resistance to erythromycin in the genus Rickettsia by mutations in L22 ribosomal protein. *J. Antimicrob. Chemother.* 56 396–398. 10.1093/jac/dki242 15996971

[B51] Rosenberg E. Zilber-Rosenberg I. (2018). The hologenome concept of evolution after 10 years. *Microbiome* 6:78. 10.1186/s40168-018-0457-9 29695294 PMC5922317

[B52] Sabaneyeva E. Castelli M. Szokoli F. Benken K. Lebedeva N. Salvetti A. (2018). Host and symbiont intraspecific variability: the case of *Paramecium calkinsi* and “*Candidatus* Trichorickettsia mobilis”. *Eur. J. Protistol.* 62 79–94. 10.1016/j.ejop.2017.12.002 29287245

[B53] Schindelin J. Arganda-Carreras I. Frise E. Kaynig V. Longair M. Pietzsch T. (2012). Fiji: an open-source platform for biological-image analysis. *Nat. Methods* 9 676–682. 10.1038/nmeth.2019 22743772 PMC3855844

[B54] Schweikert M. Fujishima M. Görtz H.-D. (2013). “Symbiotic associations between ciliates and prokaryotes,” in *The Prokaryotes*, eds Rosenberg E. DeLong E. F. Lory S. Stackebrandt E. Thompson F. (Berlin: Springer), 427–463. 10.1007/978-3-642-30194-0_18

[B55] Sekeyová Z. Danchenko M. Filipèík P. Fournier P. E. (2019). Rickettsial infections of the central nervous system. *PLoS Negl. Trop. Dis.* 13:e0007469. 10.1371/journal.pntd.0007469 31465452 PMC6715168

[B56] Serra V. Gammuto L. Nitla V. Castelli M. Lanzoni O. Sassera D. (2019). Next generation taxonomy: integrating traditional species description with the holobiont concept and genomic approaches-The in-depth characterization of a novel *Euplotes* species as a case study. *bioRxiv* [Preprint] 10.1101/666461

[B57] Shaked Y. Samra Y. Maier M. K. Rubinshtein E. (1989). Relapse of rickettsial Mediterranean spotted fever and murine typhus after treatment with chloramphenicol. *J. Infect. Dis.* 18 35–37. 10.1016/s0163-4453(89)93567-6 2915129

[B58] Shukla P. Bansode F. W. Singh R. K. (2011). Chloramphenicol toxicity: a review. *J. Med. Med. Sci.* 2 1313–1316.

[B59] Simon J.-C. Marchesi J. R. Mougel C. Selosse M.-A. (2019). Host-microbiota interactions: from holobiont theory to analysis. *Microbiome* 7:5. 10.1186/s40168-019-0619-4 PMC6330386 30635058

[B60] Siqueira M. S. Ribeiro R. M. Travassos L. H. (2018). Autophagy and its interaction with intracellular bacterial pathogens. *Front. Immunol.* 9:935. 10.3389/fimmu.2018.00935 29875765 PMC5974045

[B61] Skovorodkin I. N. (1990). A device for immobilizing small biological objects under light optical study. *Tsitologiia* 32 301–302. 2219453

[B62] Slavchev G. Michailova L. Markova N. (2016). L-form transformation phenomenon in *Mycobacterium tuberculosis* associated with drug tolerance to ethambutol. *Int. J. Mycobacteriol.* 5 454–459. 10.1016/j.ijmyco.2016.06.011 27931687

[B63] Sonntag B. Sommaruga R. (2020). Effectiveness of photoprotective strategies in three mixotrophic planktonic ciliate species. *Diversity* 12:252 10.3390/d12060252

[B64] Suchland R. J. Geisler W. M. Stamm W. E. (2003). Methodologies and cell lines used for antimicrobial susceptibility testing of *Chlamydia* spp. *Antimicrob. Agents Chemother.* 47 636–642. 10.1128/aac.47.2.636-642.2003 12543671 PMC151736

[B65] Szokoli F. Castelli M. Sabaneyeva E. Schrallhammer M. Krenek S. Doak T. G. (2016). Disentangling the taxonomy of *Rickettsiales* and description of two novel symbionts (”*Candidatus* Bealeia paramacronuclearis” and “*Candidatus* Fokinia cryptica”) sharing the cytoplasm of the ciliate protist *Paramecium* biaurelia. *Appl. Environ. Microbiol.* 82 7236–7247. 10.1128/aem.02284-16 27742680 PMC5118934

[B66] Theis K. R. Dheilly N. M. Klassen J. L. Brucker R. M. Baines J. F. Bosch T. C. G. (2016). Getting the hologenome concept right: an eco-evolutionary framework for hosts and their microbiomes. *mSystems* 1:e00028-16. 10.1128/mSystems.00028-16 PMC5069740 27822520

[B67] Trastoy R. Manso T. Fernández-García L. Blasco L. Ambroa A. Pérezdel M. M. L. (2018). Mechanisms of bacterial tolerance and persistence in the gastrointestinal and respiratory environments. *Clin. Microbiol. Rev.* 31:e00023-18. 10.1128/CMR.00023-18 PMC6148185 30068737

[B68] Van den Bergh B. Michiels J. E. Fauvart M. Michiels J. (2016). Should we develop screens for multi-drug antibiotic tolerance? *Expert Rev. Anti Infect. Ther.* 14 613–616. 10.1080/14787210.2016.1194754 27227426

[B69] Vannini C. Boscaro V. Ferrantini F. Benken K. A. Mironov T. I. Schweikert M. (2014). Flagellar movement in two bacteria of the family *Rickettsiaceae*: a re-evaluation of motility in an evolutionary perspective. *PLoS One* 9:e87718. 10.1371/journal.pone.0087718 24505307 PMC3914857

[B70] Vannini C. Petroni G. Verni F. Rosati G. (2005). *Polynucleobacter* bacteria in the brackish-water species *Euplotes harpa* (Ciliata Hypotrichia). *J. Eukaryot. Microbiol.* 52 116–122. 10.1111/j.1550-7408.2005.04-3319.x 15817116

[B71] Vannini C. Pöckl M. Petroni G. Wu Q. L. Lang E. Stackebrandt E. (2007). Endosymbiosis *in statu nascendi*: close phylogenetic relationship between obligately endosymbiotic and obligately free-living *Polynucleobacter* strains (*Betaproteobacteria*). *Environ. Microbiol.* 9 347–359. 10.1111/j.1462-2920.2006.01144.x 17222133

[B72] Vannini C. Sigona C. Hahn M. Petroni G. Fujishima M. (2017). High degree of specificity in the association between symbiotic *Betaproteobacteria* and the host *Euplotes* (Ciliophora, Euplotia). *Eur. J. Protistol.* 59 124–132. 10.1016/j.ejop.2017.04.003 28521174

[B73] Vanrompay D. Nguyen T. L. A. Cutler S. J. Butaye P. (2017). Antimicrobial resistance in *Chlamydiales*, *Rickettsia*, *Coxiella*, and other intracellular pathogens. *Microbiol. Spectr.* 6:ARBA-00032017. 10.1128/microbiolspec.arba-0003-2017 PMC11633567 29651977

[B74] Vishnyakov A. Rodionova G. (1999). “Motile intranuclear symbionts of ciliate *Paramecium multimicronucleatum*,” in *Symbiosis to Eukaryotism. Endocytobiology, VII*, eds Wagner E. Normann J. Greppin H. Hackstein J. H. P. Herrmann R. G. Kowallik K. V. (Geneva: University of Geneva), 169–177.

[B75] Walker D. (2009). The realities of biodefense vaccines against *Rickettsia*. *Vaccine* 27 (Suppl. 4), D52–D55. 10.1016/j.vaccine.2009.07.045 19837287 PMC2909128

[B76] Weissmann G. (2005). Rats, lice and Zinsser. *Emerg. Infect. Dis.* 11 492–496. 10.3201/eid1103.AD1103 15789497 PMC3298258

[B77] Wisseman C. L. Jr. Waddell A. D. Walsh W. T. (1974). In vitro studies of the action of antibiotics on *Rickettsia prowazekii* by two basic methods of cell culture. *J. Infect. Dis.* 130 564–574. 10.1093/infdis/130.6.564 4214880

[B78] Wisseman C. L. Jr. Silverman D. J. Waddell A. Brown D. T. (1982). Penicillin-induced unstable intracellular formation of spheroplasts by rickettsiae. *J. Infect. Dis.* 146 147–158. 10.1093/infdis/146.2.147 6809842

[B79] Zilber-Rosenberg I. Rosenberg E. (2008). Role of microorganisms in the evolution of animals and plants: the hologenome theory of evolution. *FEMS Microbiol. Rev.* 32 723–735. 10.1111/j.1574-6976.2008.00123.x 18549407

