# Supplementary figures and images for: A Robust Symbiotic Relationship Between the Ciliate Paramecium multimicronucleatum and the Bacterium Ca. Trichorickettsia Mobilis
Source: Front Microbiol. 2020 Nov 24;11:603335. doi: 10.3389/fmicb.2020.603335 (PMC7721670; doi:10.3389/fmicb.2020.603335)

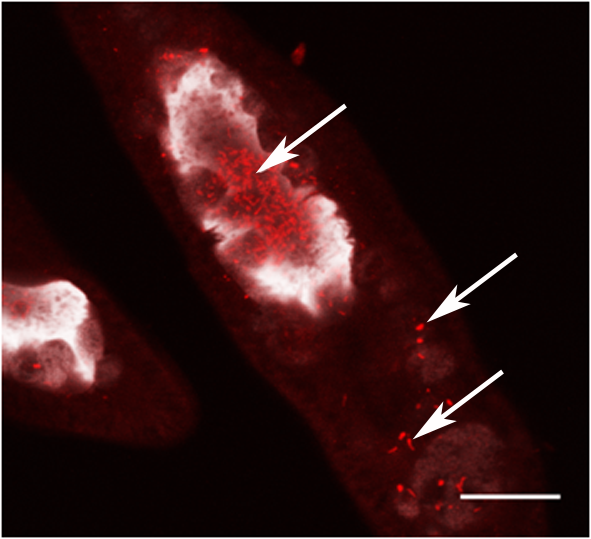

Supplement: Supplementary Figure 1 — Paramecium multimicronucleatum (strain LSA11-2) on the 3rd day after ampicillin treatment. FISH with Alphaproteobacteria specific probe Alf_1b (red signal). Arrows point to Ca. Trichorickettsia mobilis located in the macronucleus (counterstained with DAPI, shown in white) and in the cytoplasm. Scale bar, 20 μm. [file Image_1.TIF]
